# From Ambient Sensing to IoT-based Context Computing: An Open Framework for End to End QoC Management ^†^

**DOI:** 10.3390/s150614180

**Published:** 2015-06-16

**Authors:** Pierrick Marie, Thierry Desprats, Sophie Chabridon, Michelle Sibilla, Chantal Taconet

**Affiliations:** 1 IRIT UMR 5505 Université Paul SABATIER, 31062 Toulouse, France; E-Mails: thierry.desprats@irit.fr (T.D.); michelle.sibilla@irit.fr (M.S.); 2 Institut Mines-Télécom, CNRS UMR 5157 SAMOVAR, Télécom SudParis, 91011 Évry, France; E-Mails: sophie.chabridon@telecom-sudparis.eu (S.C.); chantal.taconet@telecom-sudparis.eu (C.T.)

**Keywords:** Quality of Context, quality criterion, context management, meta-modeling, information model

## Abstract

Quality of Context (QoC) awareness is recognized as a key point for the success of context-aware computing. At the time where the combination of the Internet of Things, Cloud Computing, and Ambient Intelligence paradigms offer together new opportunities for managing richer context data, the next generation of Distributed Context Managers (DCM) is facing new challenges concerning QoC management. This paper presents our model-driven QoCIM framework. QoCIM is the acronym for Quality of Context Information Model. We show how it can help application developers to manage the whole QoC life-cycle by providing genericity, openness and uniformity. Its usages are illustrated, both at design time and at runtime, in the case of an urban pollution context- and QoC-aware scenario.

## 1. Introduction

Context-aware applications become widely available and are entering our everyday lives. These applications consume context information extracted from local ambient data, user profiles, collected from heterogeneous and spatially distributed sensors. As defined by [1], we consider context information as “any information that can be used to characterize the situation of an entity. An entity is a person, place, or object that is considered relevant to the interaction between a user and an application, including the user and application themselves.” Because context information is intrinsically inaccurate, erroneous and ambiguous [2], the behavior of context-aware applications is strongly affected by the Quality of the Context information (QoC). As a consequence, QoC-aware applications require a fine and efficient management of the QoC they rely on. QoC is related to any piece of information that describes the quality of context as defined by [3]. One solution to handle the context information is to use Context Managers (CM). Context managers are software platform that support context information throughout its life cycle. The life cycle of a piece of context information starts when it gets collected by a sensor and ends with its consumption by a context-aware application. A bad quality of context could lead applications to wrong decisions and irrelevant reactions. That is why context managers must take into account QoC at each step of the context information life cycle.

According to the forecasting vision of the Internet of Things (IoT) made by [4], everyday objects will be connected to the Internet and share information with each other. Then, extending the scope of context managers from local ambient environments to the Internet of Things necessarily introduces a spatio-temporal decoupling between context producers (e.g., embedded sensors or smart objects) and context consumers (e.g., context-aware applications running on mobile devices). Consequently new challenges arise in order to guarantee the effectiveness and the efficiency of the new generation of context managers, corresponding to Distributed Context Managers (DCMs). Such DCMs must be deployed at multiple scales over various devices or servers, spread across heterogeneous networks. In addition to the classical key points used for the successful determination of the behavior of context-aware applications, QoC is essential to contribute to both the effectiveness and the efficiency of such context managers.

DCMs are distributed over the Internet. This implies that it is no more possible to establish a kind of “one-to-one QoC-based contract” between context producers and context consumers. Nevertheless, handling QoC requirements and QoC guarantees is still essential. Context consumers express QoC requirements about the quality they need and, symmetrically, context producers express QoC guarantees about their capabilities. According to the consumer's expectations, DCMs have to deliver context information with the appropriate QoC. Moreover, because one of the main functionalities of context managers is to process context information (for example: aggregation, inference and so on), they also have to manipulate QoC during the execution of these operations. In addition, DCMs should be extensible by enabling the design and the computation of any QoC criterion.

The remainder of this paper is structured as follows: Section 2 motivates the use of DCMs with a fictional scenario inspired from an existing concern: the urban pollution. The scenario illustrates four key challenges faced by the new generation of context managers. Section 3 describes our model-driven approach for QoC management and positions it with respect to related works. Divided into four parts, the architecture of a distributed context manager is then presented in Section 4 and compared to related works in the domain. Sections 5 to 8 detail these four parts through the implementation of the urban pollution scenario. Section 5 depicts how to produce context information with QoC meta-data. The context information and QoC meta-data processing is formalized in Section 6. Symmetrically, Section 7 presents how QoC-aware applications are able to interpret the QoC meta-data they receive. The fourth part of the context manager, focused on routing context information to QoC-aware applications, is described in Section 8. Finally, Section 9 summarizes our contributions to end to end QoC management within context managers.

## 2. An Urban Pollution Measurement Scenario

**Figure 1:**
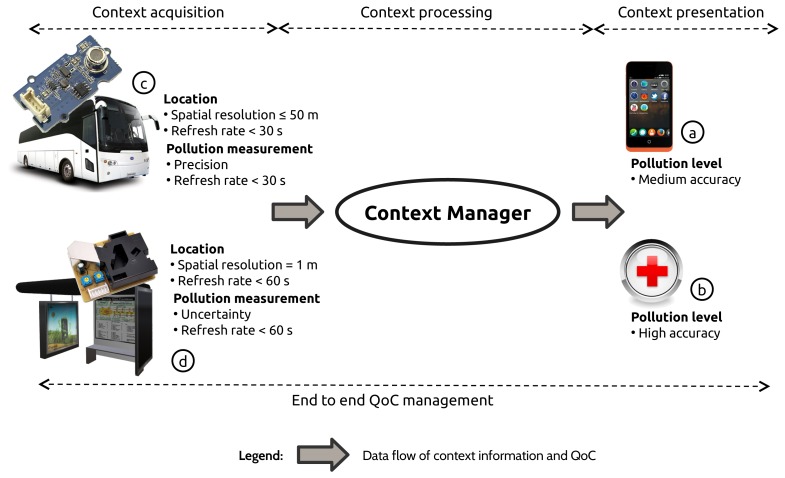
Pollution measurement scenario.

A city wants to inform its citizens in real-time about the pollution level in the streets. Inspired by the increasing citizens' concern on urban pollution, this section details the fictional scenario we use throughout the paper. Figure 1 illustrates the context producers and consumers deployed in the scenario.

Two different QoC-aware applications subscribe to context information and QoC meta-data from the context manager. Application 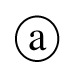
informs the citizens ans tourists about the current pollution level in the streets and helps them to optimize their travels through the city. Sensitive people like children or asthmatic people use application 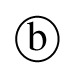, a healthcare service, to avoid any polluted street. In this part of the scenario, the pollution level is the piece of context information consumed by the applications.

The Context Manager has to offer understandable and easy-to-use context information with a high abstraction level. That is why we choose to consider the pollution level in the form of the Air Quality Index (AQI) [5] which is divided into six symbolic levels: *good, moderate, unhealthy for sensitive groups, unhealthy, very unhealthy and hazardous*. The QoC meta-data used to qualify the pollution level is made up of the *accuracy* QoC criterion used to characterize “the probability that an instance of context accurately represents the corresponding real world situation, as assessed by the context source, at the time it was determined”. This definition is used by [6] to depict the correctness criterion. This criterion possesses three symbolic labeled values: *low, medium and high. Low* means the pollution level is not reliable and *high* means the pollution level is very reliable. One difference between applications 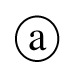
and 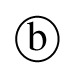 is the value of the accuracy QoC indicator they require. The general public application requires a medium QoC level whereas the healthcare application requires a high QoC level.

In order to provide QoC-aware applications with the context information they require, the city equips the public buses 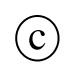
with a cheap pollution sensor [7] a GPS receiver and an embedded software. This software collects and publishes pollution measurements (example: Carbon Monoxide: 46 parts per million) and the location of the bus during the measurements. The city also equips the public bus stations 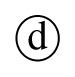 with sophisticated pollution sensors [8] and an embedded software. In this part of the scenario, the pollution measurements represent the context information. Four QoC criteria are used to qualify pollution measurements and also the GPS location.

- Uncertainty allows to “quantify doubt about the result of a measurement” [9]. The uncertainty is an interval expressed in the same unit as the measurement.
- Precision qualifies “how close together or how repeatable the results from a measurement are” [10]. Precision is expressed in percent.
- Refresh rate is “the time that elapses between the determination of context information and its delivery to a requester”. This definition is used by [3] to characterize the freshness. Because the refresh rate measures a period, its unit is the second.
- Spatial resolution corresponds to “the precision with which the physical area, to which an instance of context information is applicable, is expressed” [6]. Spatial resolution measures a distance, so its unit is the meter.

The spatial resolution is used to qualify the bus's and bus station's locations. The location and the pollution measurements are qualified with a refresh rate indicator. Finally, the precision qualifies the pollution measurements provided by the buses while the bus stations use the uncertainty to qualify their pollution measurements. Table 1 summarizes the context information and QoC meta-data produced by the embedded software deployed on the buses and the bus stations. The table also summarizes the information used by the general and healthcare application. The table highlights the necessity to apply some processing on context information and QoC meta-data forwarded by the context consumers to supply the QoC-aware applications with the appropriate abstraction level as pollution measurements should be transformed to determine the Air Quality Index (AQI).

This scenario fits three of the four domains identified by [11] concerning the futuristics IoT-based applications: smart environments, transportation and logistics and healthcare. Smart environments and the healthcare domain because the environment surrounding users will indicate where are the most polluted streets, and transportation because the applications are used to improve the travels in the city. Figure 1 illustrates the clear distinction that has to be done between context acquisition, processing and presentation. Acquisition relies on collecting raw data mostly produced by sensors. Processing is the place where transformations are applied to the data provided by the acquisition in order to supply the presentation part with the appropriate high level context information. Transversally, the QoC has to be handled within by the acquisition, processing and the presentation. As for context information, the abstraction level of the QoC meta-data increases from the acquisition to the presentation. In the scenario, two types of QoC-aware application and context producer have been identified, but some questions still remain:

1. How to formally define the quality of the information produced by the embedded softwares?
2. How to apply transformations to provide high level QoC meta-data?
3. How to formally define the quality of the information required by the applications?
4. How to route the appropriate context information with the required QoC level to the applications?

With the increasing number of producers and consumers coming from the IoT, the efficiency of the new generation of context managers strongly depends on their capabilities to answer these questions. The following sections demonstrate and illustrate how our Quality of Context Information Model (QoCIM) eases development of context managers that take into account the quality of context in a model-driven approach.

## 3. A Model Driven Approach for QoC Definition

Since [3] identified the necessity of taking into account the quality of context information more than 10 years ago, many authors proposed different lists of QoC criteria. The first part of this section illustrates the heterogeneity of the QoC criteria that have been proposed. Then, the second part explains how our solution is able to handle different definitions of QoC criteria and integrate them as QoC meta-data.

### 3.1. Related and Previous Works on QoC Criteria

The first list of QoC criteria was defined by [3]. The authors proposed to qualify context information with “precision”, “probability of correctness”, “trust-worthiness”, “resolution” and “up-to-dateness”. Like [6], they only provide a textual definition of other criteria.

The first mathematical definition of QoC criteria has been offered by [13]. [14] analyzed the criteria proposed by [3,6,13], and provided a new list of QoC criteria. They redefined “up-to-dateness”, “sensitiveness”, “access security”, “completeness”, “precision” and “resolution”. They provided either a mathematical formula or algorithms to compute the value of the criteria.

Neisse [10] suggested in 2012 to adopt the ISO standard used in metrology to define QoC. The authors analyzed the concepts of “accuracy” and “precision” as just an approximative definition of the precision defined in metrology. In the same way, the concepts of “spatial resolution” and “temporal resolution” defined by [6] match the ISO definition of the precision applied on spatial and temporal information. Finally, [10] proposed to measure QoC with only two criteria, the age of the context information and the ISO definition of the precision.

Marie [12] proposed a comparative and critical analysis of the existing QoC criteria used to qualify context information in the research works of the past decade. Table 2 is an extract of this comparison and is reminded here to justify some of the points presented further in this paper.

The criteria described in rows 1, 10 and 11 of Table 2 are possible definitions of the precision criterion presented in the pollution measurement scenario. The criteria described in rows 5, 6 and 15 in Table 2 are possible definitions of the refresh rate criterion presented in Section 2. Some criterion names are followed by numbers. For example, on line 15, the timeliness criterion defined by [15] is followed by the numbers 5 and 6. These numbers reference the numbers in the first column and indicate that the criterion is composed with other criteria. In this example, the timeliness defined by [15] is computed with the time period and the measurement time. This kind of criterion is a composite criterion.

The table illustrates that there is no consensus about the name, the meaning and the computation method used to define the QoC criteria. This supports one of the conclusions of [16] stating that providing a common definition of the criteria used to qualify context information within context managers is still an open problem. Instead of defining yet another list of QoC criteria, our approach consists of providing a meta-model able to represent any criterion which application developers can then rely on to define their own QoC criteria. The result of this approach is the Quality of Context Information Model (QoCIM) introduced in [12]. Section 3.2 highlights the key modeling elements of QoCIM.

### 3.2. Overview of the QoCIM Meta-Model

QoCIM is not dependent on any QoC criterion. It derives from interesting concepts extracted from several models studied in [12] in the form of a meta-model able to support the design and the representation of heterogeneous QoC criteria. The purpose of the QoCIM meta-model is to offer a solution to define QoC criteria that could be:

**primitive:** a criterion that does not depend on any other criteria for its definition, for example, the usability in Table 2;
**composite:** a criterion built upon other criteria, as for the timeliness criterion in Table 2;
**invariant:** a criterion that has a well defined list of possible values, for example, the accuracy criterion illustrated in the pollution scenario.

**Figure 2:**
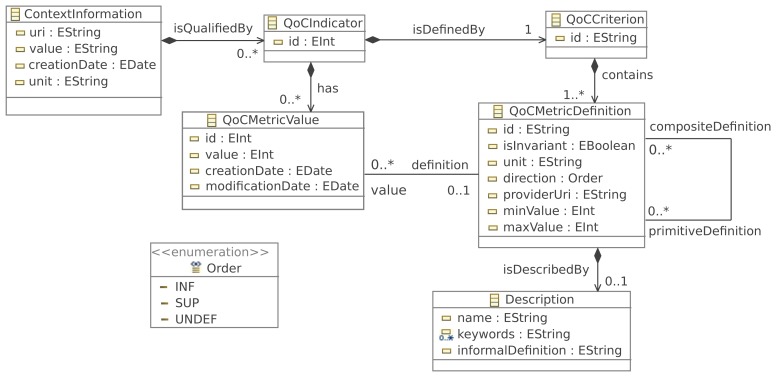
QoCIM meta-model.

Figure 2 contains, as a reminder, an overview of QoCIM. The model is built upon six interrelated constructions that are respectively dedicated to the definition and description of the QoC criterion (classes QoCCriterion, QoCMetricDefinition and Description), its valuation (class QoCMetricValue) and the binding of a QoC criterion (class QoCCriterion) with context information to be qualified (classes QoCIndicator and ContextInformation). The genericity of QoCIM mainly relies on the characteristics of some associations between these concepts. Moreover, the fields of the class ContextInformation represent the essential elements of a context information and it is still possible to integrate this class within a more complex information model as, for example, the model used by [17].

#### 3.2.1. Context Information Is Qualified by QoC Indicators

A piece of context information can be qualified by several QoC indicators. Pragmatically, we assume that QoC indicators are considered as meta-data associated with one piece of context information. The fact that the arity of this association is not constrained brings flexibility in handling only useful QoC meta-data in comparison with an approach that would impose to systematically use a mandatory list of specific QoC indicators.

#### 3.2.2. A QoC Criterion Contains QoC Metric Definitions

To one QoC criterion can be associated several metric definitions, all of these definitions are uniquely identified and characterized. This generic modeling approach gives the opportunity to describe different possible ways to obtain one metric value of a same QoC indicator. Consequently, many characterizations of a same QoC criterion may coexist within an heterogeneous system, but each one can be commonly interpreted thanks to the pivot modeling language corresponding to QoCIM. Such an approach improves the interoperability level.

#### 3.2.3. Defining Composite QoC Metric Definitions

With the recursive link on the class QoCMetricDefinition, a QoC criterion can be defined from a composition of two or more primitive criteria. This ability is helpful when a global and general indication about the QoC is required. It allows to build a high level QoC criterion that reports a symbolic level of quality. This recursive relationship contributes to the openness of the QoC modeling process by giving the ability to easily define any novel composite QoC criterion. An example of composite QoC criterion is provided by [18]. The author proposes to compute the “probability of correctness” (the probability that the provided context information is correct) with the precision, up-to-dateness and trustworthiness previously defined by [3].

The following section describes the features of our graphical editor, based on the QoCIM meta-model, enabling to define any QoC criterion class diagram.

### 3.3. A Graphical Editor to Define QoC Criteria

Our approach consists of providing developers with a graphical editor dedicated to produce, at design time, new UML class diagrams of QoC criteria. With the editor, it is possible to modify the criteria by adding new definitions, new descriptions or new QoC metric values. The editor also enables to define composite criteria depending on other already defined criteria. For that purpose, we modeled QoCIM as an Ecore model based on the EMF technology [19] and we developed a graphical editor with the Sirius technology [20].

**Figure 3:**
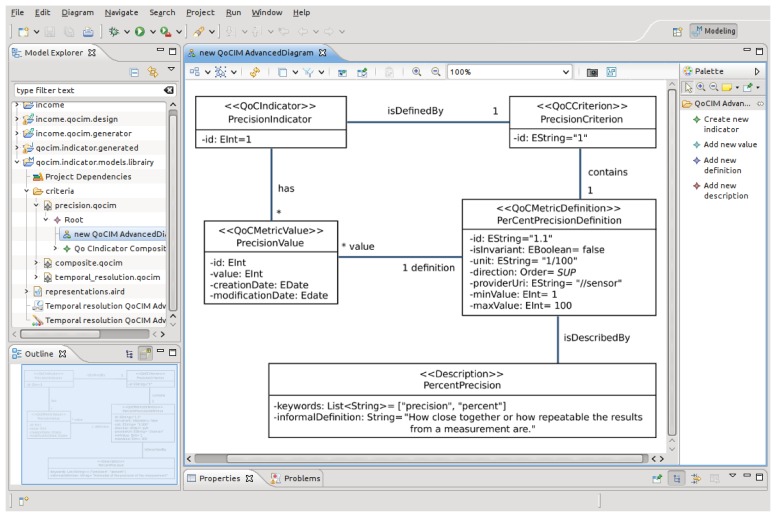
The QoCIM-based graphical editor.

Figure 3 is a screen shot of the editor. The right side of the screen contains the four essential actions used for creating new QoC indicators: “Create new indicator”, “Add new value”, “Add new definition”, “Add new description”. The main panel, in the middle of the screen, is the area used to define QoC indicators. The model presented in Figure 3 is the precision indicator. All the values of the fields presented in the model have been edited with the tab “Properties” in the bottom of the screen. The left side of the screen contains an overview of the models available in the current Eclipse project. In this example, three models are edited: “precision.qocim”, “composite.qocim”, “temporal_resolution.qocim”. All these files are XML documents where each of them defines a QoC indicator.

At programming time, developers of QoC-aware applications choose the QoC criteria they require and use the editor to generate the source code corresponding to these criteria. All the classes and fields edited in the main view of the editor are translated into Java code. Moreover, sharing the QoC meta-data with the context manager becomes easy as the QoCIM framework is able to serialize any QoC criterion into an XML document. The Acceleo technology [21] has been used to develop the module allowing to generate the source code. This kind of approach has already been explored by [22] where the authors propose a DSL to generate the Java source code corresponding to context- and QoC-aware applications. Our solution consists of substituting the DSL by a graphical editor to generate the source code of the QoC criteria.

## 4. Architecture of a Distributed Context Manager

This section highlights when and where QoC indicators may act within the architecture of a context manager. In a first time, the related works concerning existing context managers that take into account QoC are presented. Then, the integration of QoCIM within our context manager is presented in the second part of this section.

### 4.1. Related Works on Context Managers Integrating QoC

The AWARENESS project [6] identified three reasons for taking into account the QoC within context managers. These reasons are “application adaptation”, “middleware efficiency” and “users' privacy enforcement”. The authors used five indicators to define the QoC and planned to provide a formal methodology to evaluate the value of the QoC. With a formal methodology, the authors expected to clearly and unequivocally share a value of the QoC between all the actors interacting with context managers. In the solution proposed by [6] all the parties should have an agreement in advance about the QoC they provide and require before sharing their context information.

The COSMOS project [23] proposed mechanisms for QoC management. COSMOS is a context-manager used by context-aware applications to get context information. It handles the QoC provided to applications with a contract-based system. A contract is established between a context-aware application and a context information source. The contract defines the QoC level that the context-aware application requires. The COSMOS project does not provide a formal methodology to evaluate the value of the QoC as [6] expected. The definition of the QoC is still open and the developers of the context-aware applications have to specify how to evaluate the QoC within contracts. The QoC constraints expressed in a contract have to be fulfilled by the context manager during the execution of the application.

Bellavista *et al.* [16] made a comparative analysis of context data distribution solutions. The authors proposed a generic logical view of the architecture of context data distribution systems. They distinguish the context data delivery tier from the context data management tier. The context data delivery tier is focused on disseminating and routing data whereas the context data management tier is focused on the processing and the representation of data. Then, a runtime adaptation support aims to configure and reconfigure the tiers. One of the conclusions of [16] states that providing a common definition of the list of criteria used to qualify the context information within the context managers is still an open problem.

Few context managers with a QoC management capacity have already been proposed. However, either they still have to fully integrate the QoC management [6] or their definition of QoC is not formally specified [23]. One of the objectives of the INCOME project [24] is to fill these gaps by integrating a formal definition of QoC within Distributed Context Managers as well as to preserve privacy. The purpose of the project is to control the whole life cycle of context information and its associated QoC meta-data, from the production to the consumption including the processing. The project intends to handle context information coming from ambient networks, the IoT or clouds. Context information processing is distributed on heterogeneous, mobile and resource-constrained devices or servers and finally, context information is consumed both by mobile applications or fixed nodes in a cloud.

### 4.2. Functionalities of the Context Manager Developed in the INCOME Project

**Figure 4:**
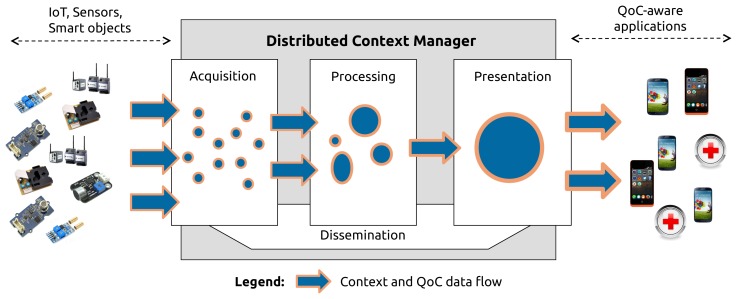
The main functionalities of a context manager.

With the increasing number of context producers, context processing entities and context consumers, a centralized solution is not feasible. That is why the new generation of context manager has to be distributed via a large collection of software entities. Some entity will be concerned by collecting raw data, then they will be deployed close to sensors. On the contrary, QoC-aware applications will be deployed closed to the users. On the middle, context processing entities can be deployed over many different machines like personal computers, dedicated servers or in the cloud. For the sequel of the article, Distributed Context Manager (DCM) designates the new generation of context manager as presented in this section. Figure 4 presents the multi-tier architecture of the context manager proposed by INCOME. It is divided into four tiers: “acquisition”, “processing”,“presentation” and “dissemination”.

#### 4.2.1. Acquisition

The acquisition tier is the access point to the context manager for receiving context information. The information is provided to the context manager by embedded software entities named *collectors*. There is no one-to-one connection between collectors and the end-user applications. The context manager has to clearly identify the capabilities of the collectors. Therefore, the context manager provides the collectors with an API to declare the type of context information and the associated QoC meta-data they supply. In a first time, collectors use the API to inform the context manager about the QoC guarantees they offer. The context manager handles two types of QoC-based constraints: (1) declaration of the QoC indicators associated to context information; (2) declaration of the QoC indicators and their value associated to context information. With the first type of constraints, there is no specification concerning the value of the indicators, only the presence of the QoC indicator is required; it is a less restrictive constraint compared to the second one that compels collectors to publish context information with a minimum QoC value. In a second time, according to their QoC guarantees, collectors share their information with the entities placed in the processing tier, via the dissemination tier, in order to produce new high level context information.

#### 4.2.2. Processing

The processing tier is handled by context processing entities deployed within the context manager to transform context data together with QoC meta-data collected from context producers. A context processing entity executes different functions like fusion, aggregation, storage, filter, inference, obfuscation to deduce new context information. As for the collectors, the context processing entities have to inform the context manager about their QoC guarantees. Then, context processing entities share their high level context information and QoC meta-data with other context processing entities or QoC-aware applications via the dissemination tier. Because context processing entities are both context producers and context consumers they also have to declare their QoC requirements before receiving information.

#### 4.2.3. Presentation

The presentation tier is the access point to the context manager for the QoC-aware applications. It works in the symmetric way from the acquisition tier. The context manager offers to context consumers an API to declare the type of context information and associated QoC meta-data they require. In a first time, the context consumers use the API to inform the context manager about their QoC requirements. For the presentation tier, the context manager handles the same type of QoC constraints as the constraints presented in the acquisition tier. In a second time, the dissemination tier is able to route the appropriate information to the consumers in push mode.

#### 4.2.4. Dissemination

The dissemination tier is the key feature of the context manager. It establishes the bindings between the acquisition, processing and presentation tiers. Dissemination consists of routing context information supplied by collectors and context processing entities to the appropriate context consumers. Context information follows the routing paths built by the dissemination from the QoC guarantees and QoC requirements expressed by the producers and consumers. In the INCOME project, the dissemination tier is handled by a Distributed Event-Based System [25].

**Figure 5:**
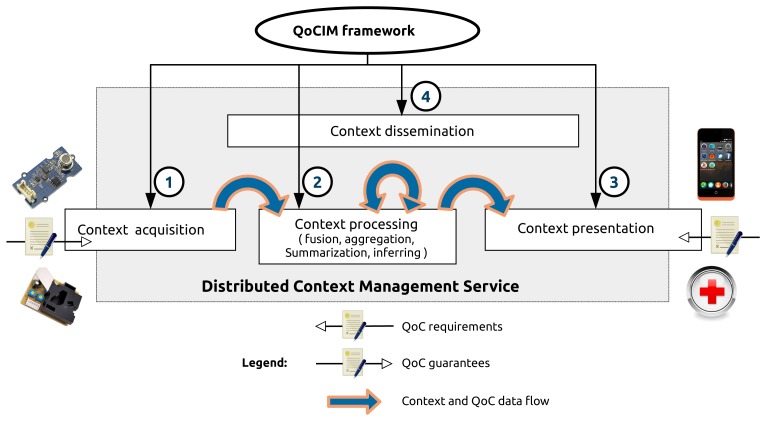
The four points where the QoCIM framework operates.

As illustrated in Figure 5, the QoCIM framework operates within the four mandatory tiers presented in Section 4.2. Based on the elements presented in Sections 3 and 4, the next sections illustrate the contributions of QoCIM through the implementation of the urban pollution scenario step by step following these tiers. Section 5 depicts the production of the information by the buses and the bus stations. The processing applied to the information collected from these context producers is described in Section 6. The QoC-aware applications are outlined in Section 7. Finally, Section 8 characterizes the filters used to route the information from collectors running on the buses and bus stations to the QoC-aware applications through different context processing entities.

## 5. Implementation of the Urban Pollution Scenario: The Acquisition Tier

The next sections detail the equations and the process used to compute the values of the QoC indicators concerning the context producers and listed in Table 1. The focus of this section is on the point ➀ of Figure 5. At programming time, the equations are implemented within the empty method getQoCMetricValue() generated by the graphical editor. Developers have to fill this method to complete the definition of the criterion with the algorithm used to get the value of the instance of the class QoCMetricValue, corresponding to a context observation. In a first time, this section focuses on the buses, the bus stations are presented in a second time.

### 5.1. The Information Published by the Bus

The following paragraphs respectively describe the implementation of the refresh rate, precision and finally the spatial resolution. An overview of the process executed by the context collector on the buses is available in Section 5.1.4.

#### 5.1.1. The Refresh Rate

The following equation computes “the time that elapses between the determination of context information and its delivery to a requester” [3]. It provides the value of the refresh rate QoC metric value.

(1) Refreshrate=currentdate−dateofmeasurement

The unit of this QoC indicator is in “seconds”. With this equation, the field *minValue* is 0 and the *maxValue* is −1, that means there is no maximum bound for this indicator. The value of the field *direction* is INF, that means the more the result of this equation increases, the more the quality of the context information decreases.

#### 5.1.2. The Precision

Based on the relative standard deviation, the value of the precision is computed with the following equation. It defines “how close together or how repeatable the results from a measurement are” [10].

(2) $$\text{precision}=\frac{\text{stantandard}\;\text{deviation}}{\text{mean}}\times 100$$

In this equation, the standard deviation and the mean are computed with the 10 last pollution measurements provided by the pollution sensor. The *unit* of this QoC indicator is “percent”. With this equation, the field *minValue* is −1. −1 is a special result used when the mean is equal to 0. Whereas the result of the equation can be more than 100, we decided to fix the maximum value of the QoC indicator to 100. As for the previous indicator, the value of the *direction* field is INF.

#### 5.1.3. The Spatial Resolution

The bus's location depends on the precision of the GPS receiver. As for the Android system [26], the GPS receiver placed in the bus provides a solution to measure the value of the spatial resolution QoC metric value. Spatial resolution denotes “the precision with which the physical area, to which an instance of context information is applicable, is expressed” [6]. It is expressed by the radius of 68% confidence. In other words, with a circle centered with the bus's latitude and longitude, and with a radius equal to the accuracy, there is a 68% probability that the true location of the bus is inside the circle. The following paragraph details the order of the actions executed by the embedded software.

The *unit* of this QoC indicator is “meter”. With this equation, the field *minValue* is −1. −1 is a special result used when the GPS receiver is not able to provide an evaluation of the spatial resolution. We decided to fix the maximum bound of the indicator to 50 m. As for the refresh rate indicator, the value of the *direction* field is INF.

#### 5.1.4. The Sequence of the Information Produced by the Collector on the Buses

**Figure 6:**
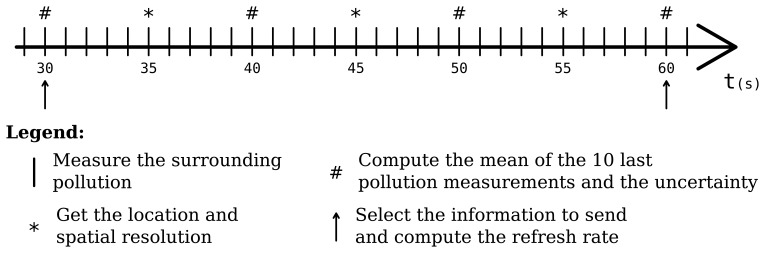
Example of pollution measurements sequence executed in a bus.

Figure 6 illustrates the four actions executed by the embedded software to produce context information and QoC meta-data:

1. every ten seconds the GPS receiver provides the bus's location and an evaluation of the spatial resolution;
2. five seconds after, the embedded software computes the mean of the ten last pollution measurements and the precision associated to the mean;
3. every thirty seconds, the embedded software selects among the three last means the value associated to the highest precision;
4. before publishing the last bus's position and the selected pollution measurement, the software completes the QoC meta-data with the estimation of the refresh rate.

### 5.2. The Information Published by the Collector on the Bus Stations

According to Table 1, the following paragraph details the last QoC indicator used by the bus stations, the uncertainty. An overview of the process executed by the context collector on the stations is available in Section 5.2.2.

#### 5.2.1. The Uncertainty

Based on the uncertainty described by [9] as a way to “quantify doubt about the result of a measurement”, the following equation details how to compute the uncertainty of the pollution measurement:

(3) $$\text{uncertainty}=\sqrt{{\left(\frac{\text{standard}\;\text{deviation}}{\sqrt{\text{nbMeasurements}}}\right)}^{2}+{\left(\frac{5\%\times \text{mean}}{\sqrt{3}}\right)}^{2}}$$

As in the previous paragraphs, the standard deviation and the mean are computed with the ten last pollution measurements made by the sensor. So, the variable *nbMeasurements* is equal to 10. The constant 5% is an arbitrary value chosen for this QoC criterion while $\sqrt{3}$ comes from the equations defined by [9]. Following the equation, the *unit* of this QoC indicator is the same as the unit of the pollution measurement, it is in parts per million (ppm). With the equation, the *minValue* field is 0 and the *maxValue* is −1, that means there is no maximum bound for this indicator. As for the refresh rate indicator, the value of the *direction* field is INF.

#### 5.2.2. The Sequence of the Information Produced by the Bus Stations

Compared to the buses, the embedded software deployed over the bus stations only executes three actions:

1. every ten seconds, the embedded software computes the mean of the ten last pollution measurements and the uncertainty;
2. every sixty seconds, the embedded software selects among the six last means the value associated to the lowest uncertainty;
3. before publishing the selected pollution measurement, the software completes the information with the bus station's position and the estimation of the refresh rate.

Concerning the production of context information and QoC meta-data, the QoCIM framework eases the definition and the implementation of QoC criteria. Then, at runtime, the QoCIM framework provides methods to transform the QoC meta-data into XML documents for the context producers to forward their information within the distributed context manager. In this section, the equations used to compute the QoC meta-data at the level of the context collectors have been clearly identified. The next section explains how these data are analyzed and transformed to provide context-aware applications with the relevant context information.

## 6. Implementation of the Urban Pollution Scenario: The Processing Tier

The first part of this section presents a state of the art of functions used to apply some processing on the context information within context managers. The conclusion of this study is a list of the most common functions used in the literature. Based on this analysis, the section then presents in a second part our definition of the functions useful for the implementation of the urban pollution scenario and their usage. The section details the point ➁ in Figure 5.

### 6.1. Related Works on Data Processing Approaches

Nurmi [27] proposed three types of operations: *pre-processing, sensor data fusion* and *context inference*. Pre-processing consists of looking for missing information and removing useless data. Sensor data fusion is the integration of multiple information measurements of the same phenomenon in a reliable way. For example, providing a single temperature from three temperature sensors when one of them is faulty calibrated. Finally, context inference deduces new high level context information from low level data.

The architecture of the context manager defined by [28] contains four modules: the *context-collector, context fusion, context-reasoner* and *context-obfuscator. Source-selector, context-fusion, high level context-extractor, context aggregator and storage* have been introduced by [15] as new keywords to describe the context manager architecture. Unfortunately, both works do not formally define their functions.

*Context data history*, *context data aggregation* and *context data filtering* are defined by [16] as three mandatory functions for the processing tier of the architecture. The context data history “captures the possibility of maintaining all relevant past events and retrieving the history of a particular context data”. Context data aggregation “provides all the fusion and merging operations capable of managing different context data”. Finally, context data filtering “strives to increase system scalability by controlling and reducing the amount of transmitted context data”.

Fanelli [29] defines the *context data storage* as the phase to “ensure context data availability and persistency”. *Context data aggregation* is the procedure used to inject new information into the context data flow. To ensure the system scalability, the *context data storage* uses different mechanisms such as caching and replication.

The definitions of *context pre-processing* and *context inference* expressed by [30] are respectively similar to the *pre-processing* and *context inference* defined by [27]. The *context data fusion* element consists of providing “more accurate, more complete, and more dependable information” impossible to obtain with a single sensor or context data source.

All the vocabulary identified in this study has been reported in the Table 3. The following sections present our formal definition of the functions used to produce the high level information for the QoC-aware applications. The required functions are filter, aggregation, and inference. The functions apply their transformations on the context information and the associated QoC meta-data. In the definitions of the functions:

- *i* represents a piece of context information and its associated QoC meta-data;
- [*i*_1_,…, *i_n_*] is a collection of pieces of information expressed with the same abstraction level, for example a collection of pollution measurements;
- *i′* a new context information resulting from the execution of a context processing function;
- *I* is a high abstract level of context information, for example a pollution level as defined by the Air Quality Index.

#### 6.1.1. Filter

The filter function is applied to a single piece of context information. The result of the function is the original piece of information or it is empty. The function does not modify the input.

(4) $$\varphi_{\text{filter}}:i,\text{condition}\mapsto \left\{\emptyset |i\right\}$$

The *condition* argument is the condition used to decide whether the input piece of information is present in the output or not. For example, if the condition is about the value of the precision indicator, then all the pieces of context information that are not associated to the expected value of the precision will not pass through the filter function.

#### 6.1.2. Aggregation

Based on the mathematical definition of the aggregation and [31], the next equation defines an aggregation function:

(5) $$\varphi_{\text{aggregation}}:\left[i_{1},\ldots ,i_{n}\right],Op_{a}\mapsto i^{\prime }$$

*Op_a_* is the aggregation operator applied to [*i*_1_*,…,i_n_*] and *i′* is the result of the operation. The aggregation creates a new piece of context information but does not change the abstraction level of the input. For example, the arithmetical mean is an aggregation operator that can be used to aggregate the pollution measurements and the precision indicator of *n* context observations to produce a new pollution measurement with a new value of the precision indicator.

#### 6.1.3. Inference

Inference is a complex function because it has to increase the abstraction level of the input information. The function uses statistical or analytical methods to produce new context information.

(6) $$F_{\text{interface}}:\left[i_{1},\ldots ,i_{n}\right],Op_{i}\mapsto I$$

In this equation, *Op_i_* is the inference operator applied to [*i*_1_,…, *i_n_*]. An example of inference function is to deduce the pollution level of a geographic area (*healthy*, *hazardous*, *dangerous* and so on) with transformation rules from different pollution measurements.

#### 6.1.4. Fusion

According to the data fusion process model defined by [32] and the state of the art made by [33], fusion is not an atomic function like filter, aggregation and inference. Fusion is the result of the composition of many atomic functions. In common definitions the first step of the fusion consists of filtering the input information to select the most one. Once the information have been cleaned some transformations can be applied as the aggregation. Simultaneously, or after, information could be stored in the purpose of using them later for statistical analysis. Finally, some inference operations can be applied to deduce high level information. The next section presents how the aggregation and inference functions provide the appropriate pollution level to context-aware applications.

### 6.2. Computing Pollution Level

As reminded in Table 1, the purpose of the functions used to produce the pollution level in a street for context-aware applications is to increase the abstraction level of the information pushed by the collectors to offer the QoC-aware applications the appropriate information. The functions used for this scenario are aggregation and inference. A context processing entity is deployed for each district of the city within the context manager and every processing entity executes these functions. The location of the pollution measurements is used to route the information to the appropriate processing entity.

#### 6.2.1. The Aggregation Function in the Pollution Scenario

An aggregation function is executed by a processing entity every five minutes to produce a new context information and QoC meta-data with the information coming from the bus stations. The operator used by the function is the geometric mean. In the same way, the arithmetical mean is used as an aggregation operator to transform the information produced by the buses. As an overview, the following equations present the signature of both functions used to aggregate the information received by the context processing entity:

(7) $$\begin{array}{l} //\text{Aggregate}\;\text{information}\;\text{coming}\;\text{the}\;\text{bus}\;\text{stations}\\ \varphi_{\text{aggregation}}:\left[\left\{\text{pollution}\;\text{measurement},\text{refresh}\;\text{rate},\text{uncertainty}\right\}\right],g\text{eometricMean}\mapsto \left\{\text{pollution}\;\text{measurement},\text{refresh}\;\text{rate},\text{uncertainty}\right\}\\ //\text{Aggregate}\;\text{information}\;\text{coming}\;\text{the}\;\text{buses}\\ \varphi_{\text{aggregation}}:\left[\left\{\text{pollution}\;\text{measurement},\text{refresh}\;\text{rate},\text{precision}\right\}\right],\text{arithmeticMean}\mapsto \left\{\text{pollution}\;\text{measurement},\text{refresh}\;\text{rate},\text{precision}\right\} \end{array}$$

##### 6.2.2. The Inference Function in the Pollution Scenario

The purpose of the inference function in the scenario is to transform the information resulting from the aggregation functions into new context information of higher abstraction level together with QoC meta-data. To realize it, the function relies upon the rule used to compute the Air Quality Index of a street and the rule used to compute the value of the accuracy QoC indicator. Both rules are based on thresholds.

For example, following the instruction of the Air Quality Index [34], if the quantity of Carbon Monoxide is up to 30 ppm the AQI is set to *hazardous* level. In the same way, if the quantity of Carbon Monoxide is less than 4 ppm, the AQI is set to *good* level.

We consider the accuracy as a composite QoC criterion, which depends on the value of all the other indicators: refresh rate, precision and uncertainty. As for the other QoC indicators presented in Section 5, the following constraints are implemented in the empty method getQoCMetricValue() of the accuracy QoC criterion generated by the graphical editor. A high accuracy value is produced if the value of the refresh rate is less than 20 s and the value of the precision is less than 10 % or the value of the uncertainty is less than 30 ppm. In the same way, a medium accuracy value is produced if the value of the refresh rate is less than 40 s and the value of the precision less than 50 % or the value of the uncertainty is less than 50 ppm. By default, a low accuracy is produced. The following equation presents the signature of the function used to infer new information coming from the bus stations and provided to the end-user applications. Concerning the information coming from the buses, the argument uncertainty is changed to precision.

(8) $$\varphi_{\text{inference}}:\left\{\text{pollution}\;\text{measurement},\text{refresh}\;\text{rate},\text{uncertainty}\right\},\text{AirQualityIndexRules}\mapsto \left\{\text{Air}\;\text{QualityIndexRules}\;\text{Index},\text{accuracy}\right\}$$

Defining functions to transform context information and QoC meta-data flows is much easier with a formal definition of QoC. It is much easier with the field direction which provides to QoCIM the ability to compare values of a same QoC indicator. For example, the well-known aggregation operator based on the mean, can be easily applied to any QoC criterion defined with the QoCIM framework. In this way, it is possible to aggregate a list of pieces of context information and QoC meta-data into a new information. Moreover, as presented in Section 3.2, QoCIM provides a way to characterize composite QoC criteria. Then, it is easy to develop functions to increase the abstraction level of QoC meta-data. The pollution level and its associated QoC level is now available for applications. The next section describes how the applications react according to the context information and the associated QoC meta-data they receive.

### 7. Implementation of the Urban Pollution Scenario: The Presentation Tier

This section shows the behavior of the general mass market application and the healthcare application according to the pollution level and the associated QoC level they receive. It corresponds to the point ➂ in Figure 5. Table 4 lists all the possible behaviors.

The first line of the table, on the top, represents the AQI pollution levels. To simplify the table, the different pollution levels are gathered two by two. The first column of the table, on the left, is the possible QoC accuracy level: high, medium and low.

The following list explains all the elements used to fill in the table:

**G:** references the General mass market application;
**H:** refers to the Healthcare application;
**usual way** is the shortest way to reach the user's destination;
**unusual way** is the recommended way to avoid the polluted streets;
**indication** is the information displayed by the application to notify the users about the dangers of the recommended way;
**warning** in warning mode, the application just recommends to find as soon as possible a safe zone delaying the travel until an unpolluted way is found.

As the composite QoC criterion has been formally defined at design time with the QoCIM framework, any other application is able to collect this high level context information and provide other behaviors or services. The next section explains how the QoCIM framework is used by the applications to express their needs in terms of QoC to the context manager. Following these needs, the section illustrates how the context manager is then able to route the appropriate context information to the right context-aware application.

### 8. Implementation of the Urban Pollution Scenario: The Dissemination Tier

After an exploration of the inside of a distributed context manager, this section presents how the dissemination tier uses the routing filters to correctly route the information. An example of such a filter specifies how a bus station provides context information and declares guarantees in terms of context information and QoC meta-data. The subject of the section is then the point ➃ in Figure 5.

**Figure 7:**
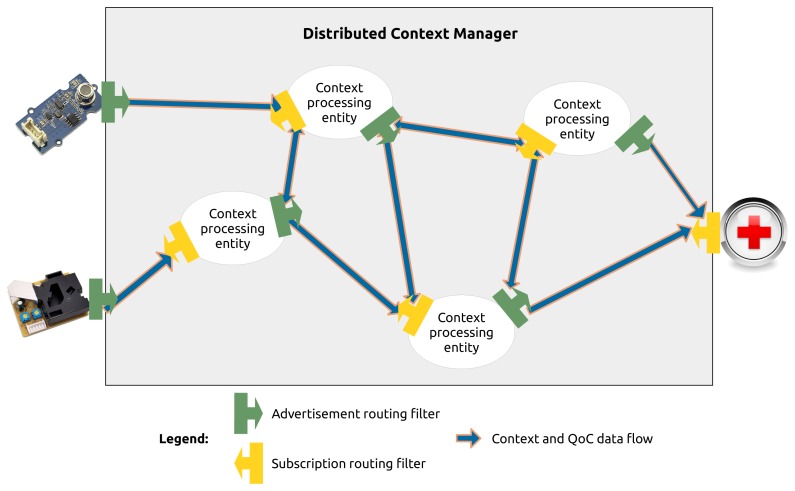
Overview of the routing filters within a distributed context manager.

Figure 7 illustrates a possible network of context processing entities used to collect, transform and produce high level context information and QoC meta-data useful for QoC-aware applications. All these processing entities do not provide the same information. Some of them could be dedicated to produce the pollution level concerning a specific geographic area whereas other entities could produce high level context information concerning all the area of the city. The challenge highlighted by the Figure is then how to correctly route context information to finally provide QoC-aware applications with the appropriate information.

Context dissemination has been already studied in [35]. The solution used to route context information is based on filters that allow, or not, to forward the information. A filter can be defined using methods offered by the API of the acquisition tier to express the QoC guarantees of the context producers and processing entities. The resulting filters are named advertisement filters. In the same way, filters are also set by the methods provided by the presentation tier to express the QoC requirements of the context processing entities and the QoC-aware applications. The resulting filters are named subscription filters.

#### 8.1. The Filters Used in the Scenario

**Figure 8:**
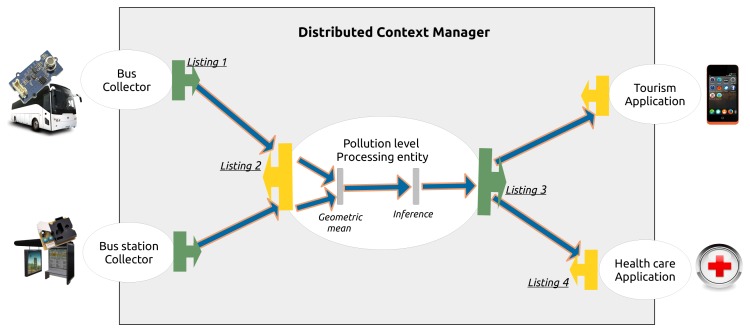
The routing filters used in the scenario to provide the pollution level of a street.

This section presents the most important routing filters used to provide the end-user applications with the current pollution of the street. More specifically, this section is focused on one context processing entity that should be dedicated to compute the pollution level of the Champs-Élysées avenue in Paris. Following this architecture, other processing entities are deployed over the other districts of the city to finally provide to the end-users the pollution level of all the main streets of the city. Figure 8 summarizes the transformations executed by the context processing entity and identifies the location of the routing filters presented in the following paragraphs.

Listing 1 details the routing filter used by the context collector placed on the bus stations to advertise the dissemination tier about its capabilities in terms of context information and QoC meta-data. [25,35] detail the engine used by the dissemination tier to handle the routing filters. Every filters are composed of a single JavaScript function named “evaluate()” and returns true or false.

Listing 1 is separated into two parts, the first part corresponds to lines 4 to 7 and the second part to lines 8 to 18. The first part is about context information. The if statement returns false when the context information does not contain an observation concerning the location expressed with the latitude and longitude and the pollution measurement expressed in particles per million. The second part of the filter is about the QoC meta-data and contains three XPath evaluations. The subject of the evaluation is the id of the QoC indicator, QoC criterion and QoC metric definitions used to discriminate all the criteria. In this example, the id refers to the first column in Table 2. Moreover, the penultimate and the last evaluations control the value of the QoC indicators. As introduced in Section 2, they respectively characterize the maximum value of the spatial resolution: 50 *m* and the refresh rate 30 *s*.


Listing 1: Advertisement filter used by the bus stations1**function evaluate**(XMLdocument) {2 importPackage(javax.xml.**xpath**);3 var XPath = XPathFactory.newInstance().newXPath();4 **if**((XPath.**evaluate**(“⫽observable[uri=‘/location’ and unit=‘WGS84’]”,5   XMLdocument, XPathConstants.NODESET).length == 0) &&6  (XPath.**evaluate**(“⫽observation[uri=‘/pollution/carbon-monoxide’ and unit=‘ppm’]”,7   XMLdocument, XPathConstants.NODESET).length == 0)) {8  **return** false;9 }10 **if**((XPath.**evaluate**(“⫽QoCIndicator[@id=‘10’ and11   QoCCriterion[@id=‘[10.1]’]/QoCMetricDefinition[@id=‘10.1’]]”,12   XMLdocument, XPathConstants.NODESET).length == 0) &&13  (XPath.**evaluate**(“⫽QoCIndicator[@id=‘7’ and14   QoCCriterion[@id=‘[7.1]’]/QoCMetricDefinition[@id=‘7.1’] and QoCMetricValue[@value<=‘50’]]”,15   XMLdocument, XPathConstants.NODESET).length == 0) &&16  (XPath.**evaluate**(“⫽QoCIndicator[@id=‘15’ and17   QoCCriterion[@id=‘[15.1]’]/QoCMetricDefinition[@id=‘15.1’] and QoCMetricValue[@value<=‘30’]]”,18   XMLdocument, XPathConstants.NODESET).length == 0)) {19  **return** false;20 }21 **return** true;22}

Listing 2 collects informations coming from the bus stations and the buses placed around the GPS location “48°52′11″N 2°18′27″E” that corresponds to the coordinate of the Champs-Élysées. The filter requires in the QoC meta-data the refresh rate QoC indicator, the spatial resolution and the uncertainty or the precision. In this way, the context processing entity is able to get the information provided by the bus stations and the buses.


Listing 2: Subscription filter used by the context processing entity1**function evaluate**(XMLdocument) {2 importPackage(javax.xml.**xpath**);3 var XPath = XPathFactory.newInstance().newXPath();4 **if**((XPath.**evaluate**(“⫽observable[uri=‘/location’ and unit=‘WGS84’]”,5   and value=‘48,52 .* N, 2,18 .* E’6   XMLdocument, XPathConstants.NODESET).length == 0) &&7  (XPath.**evaluate**(“⫽observation[uri=‘/pollution/carbon-monoxide’ and unit=‘ppm’]”,8   XMLdocument, XPathConstants.NODESET).length == 0)) {9  **return** false;10 }11 **if**((XPath.**evaluate**(“⫽QoCIndicator[@id=‘10’ and12   QoCCriterion[@id=‘[10.1]’]/QoCMetricDefinition[@id=‘10.1’]]”,13   XMLdocument, XPathConstants.NODESET).length == 0) &&14  (XPath.**evaluate**(“⫽QoCIndicator[@id=‘7’ and15   QoCCriterion[@id=‘[7.1]’]/QoCMetricDefinition[@id=‘7.1’]]”,16   XMLdocument, XPathConstants.NODESET).length == 0) &&17  ( (XPath.**evaluate**(“⫽QoCIndicator[@id=‘15’ and18   QoCCriterion[@id=‘[15.1]’]/QoCMetricDefinition[@id=‘15.1’]]”,19   XMLdocument, XPathConstants.NODESET).length == 0) ‖20  (XPath.**evaluate**(“⫽QoCIndicator[@id=‘18’ and21   QoCCriterion[@id=‘[18.1]’]/QoCMetricDefinition[@id=‘18.1’]]”,22   XMLdocument, XPathConstants.NODESET).length == 0) ) {23  **return** false;24 }25 **return** true;26}

Listing 3 is the advertisement filter of the context processing entity. It specifies the information produced by the entity. They contain an estimation of the pollution level of a street identified by its name and the QoC meta-data provide an evaluation of the accuracy QoC indicator. As for the previous routing filters, the constraint relative to the QoC meta-data relies on the id of the indicator.


Listing 3: Advertisement filter used by the context processing entity1**function evaluate**(XMLdocument) {2 importPackage(javax.xml.**xpath**);3 var XPath = XPathFactory.newInstance().newXPath();4 **if**((XPath.**evaluate**(“⫽observable[uri=‘/location’ and unit=‘title’]”,5   and value=‘Champs-Elysees’6   XMLdocument, XPathConstants.NODESET).length == 0) &&7  (XPath.**evaluate**(“⫽observation[uri=‘/pollution-level’ and unit=‘AQI’]”,8   XMLdocument, XPathConstants.NODESET).length == 0)) {9  **return** false;10 }11 **if**((XPath.**evaluate**(“⫽QoCIndicator[@id=‘17’ and12   QoCCriterion[@id=‘[17.1]’]/QoCMetricDefinition[@id=‘17.1’]]”,13   XMLdocument, XPathConstants.NODESET).length == 0) ) {14  **return** false;15 }16 **return** true;17}

Finally, Listing 4 is the subscription filter used by the healthcare application. It requires all the pollution levels available for the streets of the city associated to an accuracy level equals to high.


Listing 4: Subscription filter used by the healthcare application1**function evaluate**(XMLdocument) {2 importPackage(javax.xml.**xpath**);3 var XPath = XPathFactory.newInstance().newXPath();4 **if**((XPath.**evaluate**(“⫽observable[uri=‘/location’ and unit=‘title’]”,5   XMLdocument, XPathConstants.NODESET).length == 0) &&6  (XPath.**evaluate**(“⫽observation[uri=‘/pollution-level’ and unit=‘AQI’]”,7   XMLdocument, XPathConstants.NODESET).length == 0)) {8  **return** false;9 }10 **if**((XPath.**evaluate**(“⫽QoCIndicator[@id=‘17’ and11   QoCCriterion[@id=‘[17.1]’]/QoCMetricDefinition[@id=‘17.1’] and QoCMetricValue[@name=‘High’]]”,12   XMLdocument, XPathConstants.NODESET).length == 0) ) {13  **return** false;14 }15 **return** true;16}

Based on the QoC meta-data, the routing filters are the key elements allowing to forward the appropriate context information to the right QoC-aware applications. Because the QoCIM framework automatically generates those kinds of filters from instances of QoC indicators, it becomes much easier to set, control and keep an overview of the filters deployed over all the entities within the distributed context manager.

### 9. Conclusions

Although several works on QoC modeling and management have been conducted over the past decade, no consensual proposition has emerged. This article illustrates QoCIM, the QoC Information Model we proposed, as a generic, expressive and computable QoC information model to be used at any time during the QoC life-cycle management. In addition, the source code of the QoCIM framework is now available [36] as Maven modules [37]. As a conclusion, Table 5 summarizes the key features of the QoCIM framework and points out which part of the source code provides the feature.

QoCIM is dedicated to handle any QoC criterion within distributed context managers and QoC-aware applications. It is able to qualify a piece of context information with different QoC criteria. The same QoC criterion can be reused to qualify different pieces of context information. Sharing the definition of QoC based on the same core concepts, QoCIM provides context producers and consumers with a common language to express their QoC requirements and QoC guarantees. In this way, DCMs are able to match the needs of these producers and consumers and to evaluate QoC all along the life cycle of context information. Based on our graphical editor, the software tool chain we provide facilitates the development of QoC-aware applications. It offers a solution to easily create and use a collection of definitions of QoC criteria derived from QoCIM. Finally, the QoCIM framework supports an efficient routing system based on filters involving QoC meta-data.

## Figures and Tables

**Table 1:** Abstract of the context information and QoC meta-data used in the scenario.

		**Context Information**	**QoC Meta-Data**
*Context consumer*	**General application 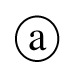 **	Pollution level (AQI)	Accuracy (symbolic label)

	**Healthcare application 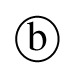 **	Pollution level (AQI)	Accuracy (symbolic label)

*Context producer*	**Buses 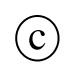 **	Pollution measurements (ppm)	Refresh rate (s); Precision (%); Spatial resolution (m)

	**Bus stations 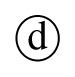 **	Pollution measurements (ppm)	Refresh rate (s); Uncertainty (ppm); Spatial resolution (m)

**Table 2:** Extract of the comparison of different lists of QoC criteria [12].

		**buchholz 2003** [3]	**kim 2006** [13]	**sheikh 2007** [6]	**filho 2010** [14]	**manzoor 2012** [15]	**neisse 2012** [10]
1	Probability context is free of errors	**Correctness**	Accuracy		**Precision**	Accuracy	**Precision**

5	Time between production of contexts			*Temporal resolution*	✓	*Time period*	

6	Date of collection of context	✓	✓	✓	✓	*Measurement time*	*Timestamps*

10	Closeness, Repeatability of measurements (ISO)						**Precision**

11	Granularity (detail level) of context	**Precision**		**Precision**	*Sensitiveness*	*Usability*	

15	Validity of context based on freshness	**Up todateness**(6)	**Up todateness**(6)	*Freshness* (6)	**Up todateness**(5, 6)	*Timeliness* (5, 6)	

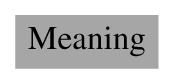
 Meaning used by all authors; *Name:* Name only defined by one author; **Name:** Name defined by different authors for different meanings; Name: Name defined by different authors for the same meaning; Name (X): The definition of this criterion depends on the X criterion; ✓*:* Criterion not defined by author but another criterion depends on it.

**Table 3:** Context processing terminology.

	**Fusion**	**Aggregation**	**Storage**	**Filter**	**Pre-Treatment**	**Inference**	**History**	**Reasoner**	**Obfuscation**	**Extraction**
[27]	✓				✓	✓				
[28]	✓							✓	✓	
[15]	✓	✓	✓							✓
[16]		✓		✓			✓			
[29]		✓	✓	✓						
[30]	✓				✓	✓				
**Occurrences**	4	3	3	2	2	2	1	1	1	1

**Table 4:** Behavior of the applications following the context information and QoC meta-data.

	***Air Quality Index***		

***Accuracy QoC Criterion***	**Good** *or* **Moderate**	**Unhealthy**	**Very Unhealthy** *or* **Hazardous**
**High accuracy**	G: usual way	G: usual way + indication	G: unusual way
	H: usual way	H: unusual way	H: unusual way

**Medium accuracy**	G: usual way + indication	G: usual way + indication	G: unusual way + indication
	H: usual way + warning	H: unusual way + warning	H: warning

**Low accuracy**	G: usual way + warning	G: unusual way + warning	G: unusual way + warning
	H: warning	H: warning	H: warning

**Table 5:** The benefits of the QoCIM framework within a Distributed Context Manager.

**General Purpose**	**QoCIM Framework Feature**

	***Application to the Pollution Scenario***
Modelling QoC criteria	At design time, developers use the graphical editor to define primitive or composite QoC criteria.

	*With the editor, defining the refresh rate, precision, spatial resolution, uncertainty and accuracy QoC indicators separately. Then, configure the relations between the accuracy definition and the uncertainty and precision*.

Qualifying context information	At programming time, developers use the editor to generate and complete the source code corresponding to the QoC criteria they choose for their applications.

	*Fill-in the method getQoCMetricValue() with the algorithm described in Section 5.1 and 5.2. (Class QoCMetricDefinition in the Maven module named qocim-common)*

Processing QoC meta-data	Because all the QoC criteria are based on the same meta-model, QoCIM eases the implementation of generic QoC transformation functions.

	*Implementing the algorithm described in Section 6.2 with the functions available in the Maven module named qocim-functions*.

Expressing QoC guarantees	The QoCIM framework provides methods to transform instances of QoC indicators into constraints that reflect the capabilities of a producer in terms of QoC.

	*In the Maven module qocim-routing-filter, using instances of the QoC indicators as parameters of the methods addQoCCriterionConstraints and addQoCValueConstraints in the class IQoCIMRoutingFilterGenerator*

Expressing QoC requirements	The QoCIM framework provides methods to transform instances of QoC indicators into constraints that reflect the needs of a consumer in terms of QoC.

	*In the Maven module qocim-routing-filter, using instances of the QoC indicators as parameters of the methods addQoCCriterionConstraints and addQoCValueConstraints in the class IQoCIMRoutingFilterGenerator*
